# Visualizing spinal tumors: a review of intraoperative contrast enhanced ultrasound during spinal tumor resections

**DOI:** 10.3389/fonc.2026.1742835

**Published:** 2026-02-27

**Authors:** Kyle P. Stegmann, Brian Fabian Saway, Liz Iglesias, Parker Dhillon, Jackson Maradik, Stephen Kalhorn

**Affiliations:** 1 College of Medicine, Medical University of South Carolina, Charleston, SC, United States; 2 Department of Neurosurgery, Medical University of South Carolina, Charleston, SC, United States

**Keywords:** contrast-enhanced ultrasound, intraoperative imaging, neuro-oncology, spinal tumors, surgical resection

## Abstract

**Objective:**

Contrast-enhanced ultrasound (CEUS) is an imaging tool that has recently gained popularity for neurosurgical applications. By using microbubble contrast agents, CEUS improves the real-time visualization of vascular structures and tissue perfusion patterns in the brain and spinal cord. Visualizing tumor margins and vascular structures is an essential aspect of spinal cord tumor resections. However, current intraoperative methods are limited. CEUS offers a dynamic, noninvasive approach to delineate margins and vascular characteristics, potentially guiding more effective tumor resection. The objective of this review is to evaluate the current body of literature on the applications and advantages of CEUS for spinal cord tumor resections.

**Methods:**

PubMed, SCOPUS, and Cochrane databases were searched for studies pertaining to CEUS utilized during spinal tumor resections. Two authors extracted and independently reviewed the data to reduce bias. Tumor type, tumor location, and primary findings were summarized.

**Results:**

Eight studies with 76 subjects were analyzed. CEUS was used in the resection of 74 intradural intramedullary and 2 intradural extramedullary tumors, all of varying tumor types. CEUS proved to be a valuable intraoperative tool for visualizing tumor margins and illustrating perfusion characteristics of intramedullary spinal tumors in real-time.

**Conclusion:**

Current literature suggests that CEUS has the capability to provide real-time data for the visualization of tumor margins and perfusion patterns of spinal cord tumors. However, with only case reports and case series currently published, there is a need for future investigation into the clinical potentials of CEUS as an adjunct tool for guiding spinal cord tumor resections.

## 1 Introduction

Spinal cord tumors, which include intradural-intramedullary, intradural-extramedullary, and extradural lesions, represent a diverse group of central nervous system (CNS) neoplasms. Notably, intramedullary tumors account for 2-4% of all primary CNS tumors (1, 2). Within the intramedullary category, common entities include subependymomas, astrocytomas, hemangioblastomas, gangliogliomas, and CNS lymphomas, with ependymomas and astrocytomas being the most prevalent in adults and children, respectively (2). These tumors often infiltrate the vasculature and parenchyma of the spinal cord, and if left untreated, can lead to decreased functional ability and reduced quality of life (3). The current standard of care for initial treatment of these neoplasms is maximal gross total resection (GTR); however, the infiltrative nature of these tumors makes it challenging to visualize margins during surgical resection, often resulting in incomplete resections, residual tumor, recurrence, and even injury to spinal cord parenchyma (4, 5). Intraoperative magnetic resonance imaging (iMRI) is currently the gold-standard to guide GTR, but is limited by the cost, additional time added to the operation, and potential artifacts from cerebral spinal fluid (CSF) and tissue shifts (6–8). Fluorescence techniques with 5-aminolevulinic acid and fluorescein have also been utilized to improve tumor margin visualization. However, this technique presents challenges, as 5-aminolevulinic acid and fluorescein only act on the visual surface of tumors. Surgeon perception can also affect visualization of tumor margins and patients must be carefully selected to avoid hypersensitivity reactions (9, 10). Given these limitations, there is currently a demand for a cheaper and quicker dynamic intraoperative tool to effectively visualize tumor margins in real-time.

Contrast-enhanced ultrasound (CEUS) may fit this demand as intraoperative ultrasound imaging offers portable and cost-effective options for visualizing pathologies in real-time (11). CEUS imaging improves conventional B-Mode ultrasound by intravenously administering a contrast agent comprised of microbubbles in a lipid casing. The highly echogenic contrast agent generates clear margins from the surrounding tissue and improves visualization of the vasculature as well as oncologic perfusion patterns that are often distinct from surrounding spinal cord (12). This allows for monitoring of vascular changes intraoperatively, identification of adjacent anatomical structures and tumor remnants, and identification of feeder arteries and veins with combined use of color doppler (13).

In the past few decades, CEUS has proven useful for visualizing brain tumors during GTR (14–16). In light of these successes and given its demonstrated ability to enhance tumor delineation and guide resection, we hypothesize that intraoperative CEUS may similarly benefit the visualization and resection of spinal cord tumors. The aim of this review is to assess the current applications and potential advantages of intraoperative CEUS in spinal oncology.

## 2 Methods

### 2.1 Search criteria

**Figure 1:**
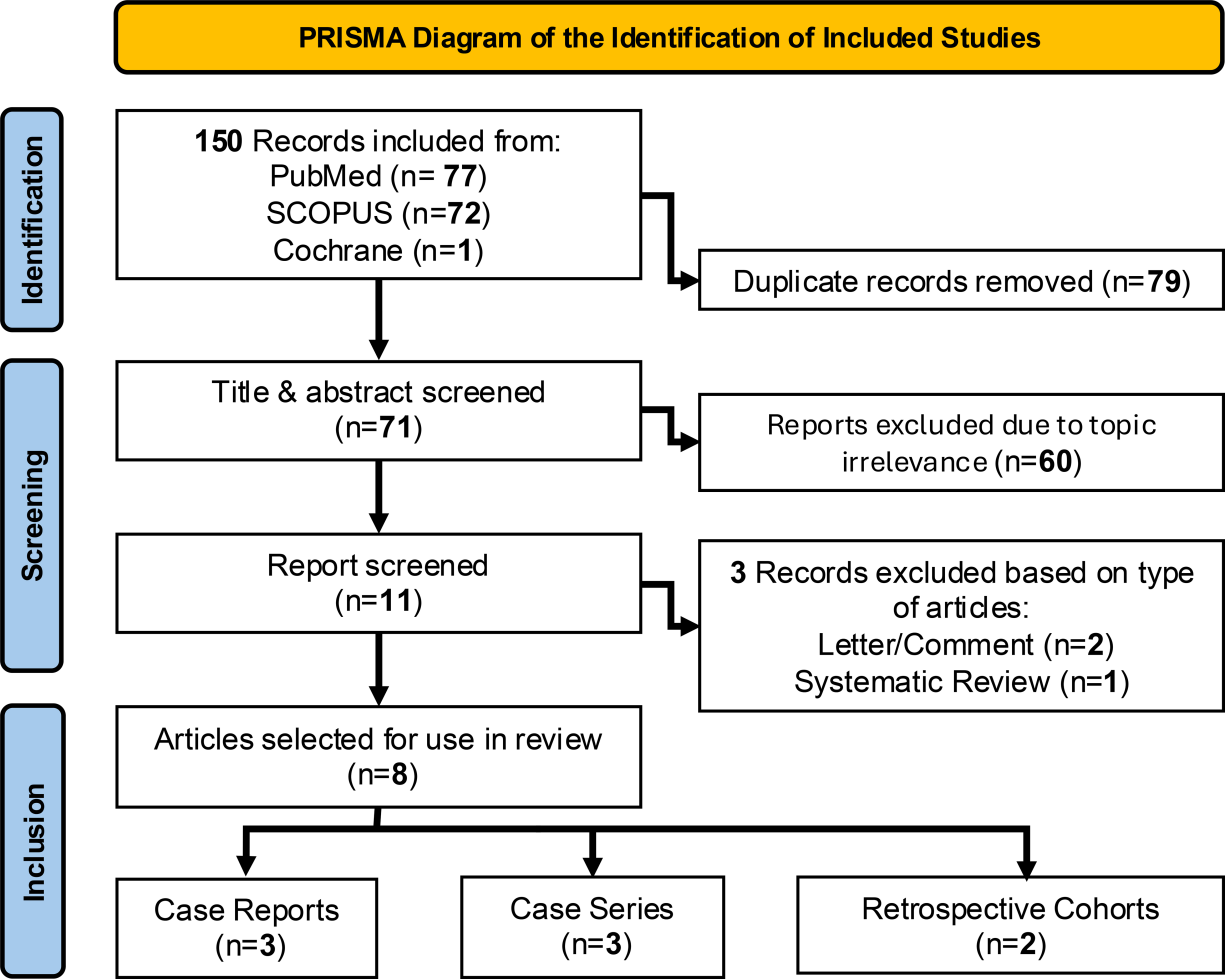
PRISMA flow diagram illustrating the database searches, screened records, and included studies for review.

This review was written following PRISMA 2020 guidelines for scoping reviews (17). Databases searched included PubMed, SCOPUS, and Cochrane. The search terms included: “CEUS AND spine”, “CEUS AND spinal tumor”, “CEUS AND spinal cord”, “CEUS AND intramedullary spinal neoplasm”, “iCEUS AND spine”, “iCEUS AND spinal tumor”, “iCEUS AND spinal cord”, “iCEUS AND intramedullary spinal neoplasm”. The database search started in late December 2024 cand continued into late March 2025, with the last database search occurring on March 20^th^, 2025. Articles were found using the keywords listed in each database, after which duplicates were excluded. Articles were then screened for their relevance to CEUS in spinal tumor resections. After the screening process, letters to the editor, conference comments, and systematic reviews were deemed ineligible. Due to the novelty of the subject, case reports and case series were included. English as a primary language was a requirement for selection. Eligibility criteria were not affected by the article’s publishing date. The PRISMA flowchart diagram illustrates our search, screening, and inclusion of reports (**Figure 1**).

### 2.2 Eligibility of articles

Articles were screened for their relevance to the topic of CEUS use in spinal tumor resections regarding human or animal participants. Excluded articles predominately highlighted non-oncological applications of CEUS, systemic oncological applications of CEUS, and standard or doppler ultrasound imaging of spinal tumors. The screening process included two authors in an independent review. Disagreement on article selection was discussed and, if needed, a third author was consulted to mitigate bias in the final selection.

### 2.3 Data collection and quality assessment

Data collection was performed by four authors. Articles were examined for the study type, tumor pathology, tumor location, and primary intraoperative CEUS findings. Enhancement characteristics, perfusion patterns, ease of delineation, and vascular quality for each tumor pathology were also recorded. Joanna Briggs Institute (JBI) assessment tools for case reports, case series, and cohort studies were used to evaluate the quality of studies and the risk of bias (18). Due to the limited quantitative data and heterogeneity of data reported in the included studies, the data analysis remained qualitative. No formal quantitative analysis was able to be performed.

## 3 Results

### 3.1 Search results and study characteristics

Of the 150 articles found in our literature search, 71 of them were unique. Once the articles were assessed for relevance to CEUS and spinal tumors, 11 reports remained for screening. We were left with 8 papers – 3 case reports, 3 case series, 2 retrospective cohort studies – after systematic reviews and comments and letters to the editor were removed (**Table 1**). A total of 76 tumors were resection with CEUS guidance, 74 of which were intradural intramedullary and 2 were intradural extramedullary. No extradural tumors were reported in the literature. Tumor type varied among all studies. The three most common types were ependymomas (29), hemangiomas (10), and glioblastomas (8).

**Table 1:** Summary of available literature on contrast-enhanced ultrasound (CEUS) and the current use within spinal tumor resection.

Author	Study type	Participants/tumor types (Total)	Tumor location(s)	Primary findings
Vetrano et al., 2015 (19)	Case Report	1 Hemangioblastoma	Intradural Intramedullary	CEUS improved visualization of tumor vascularity to allow for surgeons to better characterize the tumor and intraoperatively correct the preoperative diagnosis made by MRI.
Han et al., 2020 (25)	Case Series	4 Astrocytomas, 5 Ependymomas, 1 Anaplastic Astrocytoma, 4 Cavernous Hemangioma (14)	Intradural Intramedullary	CEUS guided localization of the lesions to determine the extent of durotomy and myelotomy, which reduced overall surgical invasiveness. CEUS also allowed for tumor margin visualization and provided information on perfusion characteristics of the lesions.
Barkley et al., 2021 (9)	Case Series	3 Hemangioblastoma, 1 Pilocytic Astrocytoma, 2 Ependymomas, 1 Subependymoma (7)	Intradural Intramedullary	CEUS improved visualizations in all cases and aid in detecting residual tumors during gross tumor resection.
Vetrano et al., 2021 (3)	Case Series	2 Pilocytic Astrocytomas, 1 Anaplastic Astrocytoma, 1 Glioblastoma, 1 Subependymoma, 4 Ependymomas, 2 Hemangioblastomas, 1 Neurocytoma (12)	Intradural Intramedullary	CEUS effectively visualized tumors in all cases and improved border definition in all but glioblastomas. Visualization of highly vascularized areas in glioblastomas aided surgical guidance for biopsies.
Mazzapicchi et al., 2022 (16)	Retrospective Cohort Study	4 Hemangioblastomas	Intradural Intramedullary	CEUS aided with surgical approach and used to described perfusion patterns.
Han et al., 2023 (26)	Retrospective Cohort Study	1 Pilocytic Astrocytoma 7 Low-Grade Gliomas, 3 Anaplastic Gliomas, 7 Glioblastomas, 18 Ependymomas (36)	Intradural Intramedullary	CEUS was able to visualize borders, vascularity, and different phases of enhancement based on tumor grade. Contrast enhancing patterns of tumors by CEUS corresponded, or improved, compared to patterns on MRI.
Vetrano et al., 2015 (20)	Case Report	1 Schwannoma	Intradural Extramedullary	CEUS further highlighted the lesion and borders to allow for differentiation from the non-enhanced spinal cord, better characterizing what was preoperatively found by MRI to be intramedullary as extramedullary.
Della Pepa et al., 2020 (13)	Case Report	1 Hemangiopericytoma	Intradural Extramedullary	CEUS allowed for the dynamic intraoperative visualization of tumor venous drainage and main arterial supply to aid in tumor resection.

### 3.2 CEUS tumor features

**Figure 2:**
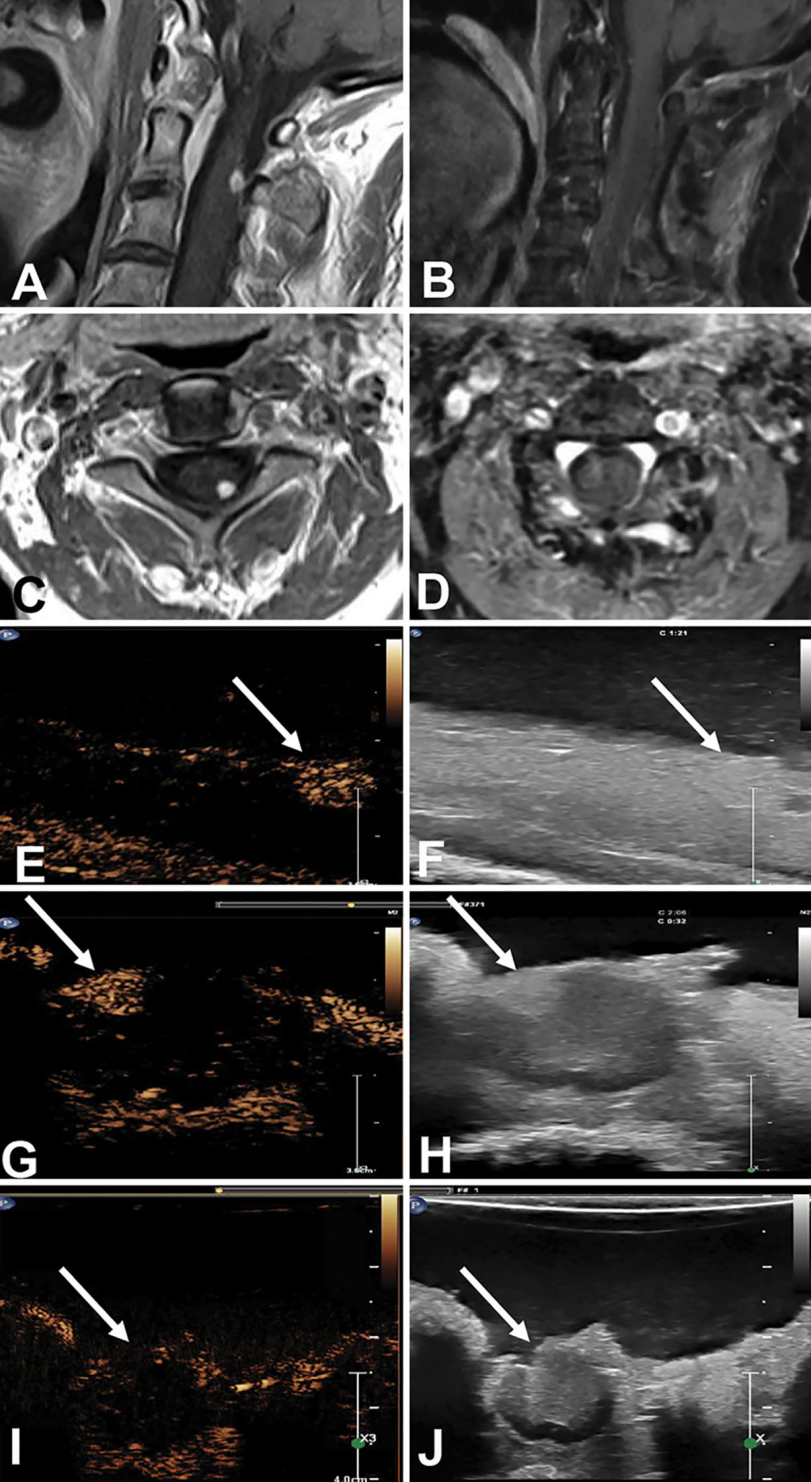
Comparison of standard B-mode ultrasound and Contrast-Enhanced Ultrasound (CEUS) in a spinal intramedullary tumor. **(A-D)** Preoperative MRI and B-mode imaging. **(E, G, I)** CEUS imaging reveals a rapidly enhancing, well-circumscribed vascular lesion (arrow), allowing for precise differentiation from the surrounding spinal cord, whereas **(F, H, J)** standard B-mode imaging demonstrates a hyperechoic lesion with less distinct margins. Reprinted from Barkley et al. (2021) [9], distributed under the terms of the Creative Commons Attribution-NonCommercial-NoDerivatives 4.0 International License (CC BY-NC-ND 4.0).

Enhancement characteristics, margin delineation, and perfusion pattern were summarized for each tumor pathology (**Table 2**). As illustrated in **Figure 2**, CEUS provides superior distinction of tumor boundaries compared to standard B-mode imaging. Well-defined borders were observed in the following pathologies: ependymomas, subependymomas, astrocytomas, pilocytic astrocytomas, neurocytoma, hemangioblastoma, hemangiopericytoma, and schwannomas. Poorly defined or blurry borders were seen with low grade gliomas, anaplastic gliomas, glioblastomas, cavernous hemangiomas, and anaplastic astrocytomas. Enhancement intensity ranged from homogenous in hemangioblastomas and hemangiopericytomas to variable in ependymomas, pilocytic astrocytomas, and subependymomas. Heterogenous enhancement was observed with hemangiopericytomas and glioblastomas, while cavernous hemangiomas showed minimal or absent enhancement. Dotted, speckled patterns were observed with ependymomas, subempendymomas, schwannomas, and low-grade gliomas. Perfusion time also varied by pathology, with rapid peak enhancement noted in hemangioblastomas, hemangiopericytomas, anaplastic astrocytomas, and glioblastomas. Slower perfusion time was observed anaplastic gliomas, pilocytic astrocytomas, subependymomas, schwannomas, neurocytomas, and low-grade gliomas. Notably, perfusion time within ependymomas varied. Vascular structures, including feeder arteries, draining veins and peritumoral cysts, were most frequently visualized in hemangioblastomas, hemangiopericytomas, ependymomas, pilocytic astrocytomas, anaplastic astrocytomas, and glioblastomas.

**Table 2:** Reported imaging characterization and perfusion patterns of tumor pathology on contrast-enhanced ultrasound (CEUS).

Pathology	n	Tumor delineation	Enhancement characteristics	Perfusion pattern	Vascularity and cyst visibility
Hemangioblastoma (WHO I)	10	Well-defined borders	Homogeneous enhancement, defined nodules	Rapid peak enhanced	Variable visibility of feeding arteries, draining veins, and peritumor cysts
Cavernous Hemangioma	4	Poor delineation	Not enhanced	–	–
Astrocytoma	4	Well-defined borders	–	–	–
Anaplastic Astrocytoma (WHO III)	2	Blurred borders	Enhancement	Rapid peak enhancement	Higher degree of vascularity
Anaplastic Glioma (WHO III)	3	Blurred borders	Homogeneous enhancement	Slower peak enhancement	Increased vascularization compared with surrounding parenchyma
Pilocytic Astrocytoma (WHO I)	4	Well-defined borders	Homogeneous, variable enhancement, centrifugal pattern	Slower peak enhancement	Feeder arteries, tumoral cysts, and syrinx visualized
Ependymomas (WHO II)	29	Well-defined borders	Variable enhancement, dotted pattern	Variable peak enhancement – slow and rapid	Increased vascularization compared with surrounding parenchyma
Subependymomas (WHO I)	2	Well-defined borders	Homogeneous aspects, dotted pattern caused by micro- and macrocysts	Slower peak enhancement	–
Schwannoma	1	Well-defined borders	Homogeneous aspects, dotted pattern	Slower peak enhancement	No visibility of peritumor cysts
Hemangiopericytoma	1	Well-defined borders	Heterogenous enhancement	Rapid enhancement	Feeder arteries and draining veins visualized
Glioblastoma (WHO IV)	8	Blurred borders	Heterogeneous, infiltrative pattern, higher degree of vascularity	Rapid enhancement	Increased vascularization compared with surrounding parenchyma
Neurocytoma (WHO II)	1	Well-defined borders	Homogeneous aspects	Slower peak enhancement	–
Low-Grade Glioma	7	Unclear borders	Mild, homogenous, dotted pattern	Slower peak enhancement	Feeder arteries not visible

### 3.3 Risk of bias assessments

JBI appraisal tools for case reports, case series, and cohort studies were used to evaluate the quality of the included studies (**Table 3**). The mean (SD) scores for the case reports and case series 7.0/8 (0.00) and 8.3/10 (0.47), respectively, indicating good methodologic quality. Case reports were limited in their inclusions of potential adverse effects, even though no adverse effects were reported. Case series were unclear regarding their description of patient inclusion, and some lacked quantitative analyses. Conversely, the mean (SD) score for the cohort studies was 5.0/11 (0.00), indicating low-moderately quality. These studies were restricted by their single-arm nature, which limited the applicability of group selection and controlling for confounding variables. Additionally, short follow-up lengths of less than 3 months limited the ability to evaluate long-term neurological recovery. Due to the limited reports of the CEUS for spinal tumor resection, the cohort studies were elected to be included.

**Table 3:** JBI case report, case series, and cohort appraisal summary.

JBI case reports appraisal	Vetrano et al., 2015 (19)	Vetrano et al., 2015 (20)	Della Pepa et al., 2020 (13)
Were patient’s demographic characteristics clearly described?	Yes	Yes	Yes
Was the patient’s history clearly described and presented as a timeline?	Yes	Yes	Yes
Was the current clinical condition of the patient on presentation clearly described?	Yes	Yes	Yes
Were diagnostic tests or assessment methods and the results clearly described?	Yes	Yes	Yes
Was the intervention(s) or treatment procedure(s) clearly described?	Yes	Yes	Yes
Was the post-intervention clinical condition clearly described?	Yes	Yes	Yes
Were adverse events (harms) or unanticipated events identified and described?	No	No	No
Does the case report provide takeaway lessons?	Yes	Yes	Yes
Total score (out of 8 points) (Yes = 1, No = 0, Unclear = 0.5, N/A = 0)	7.0	7.0	7.0

*JBI Case Series Appraisal*	Han et al., 2020 (25)	Barkley et al., 2021 (9)	Vetrano et al., 2021 (3)
*Were there clear criteria for inclusion in the case series?*	Yes	Yes	Yes
Was the condition measured in a standard, reliable way for all participants included in the case series?	Yes	Yes	Yes
Were valid methods used for identification of the condition for all participants included in the case series?	Yes	Yes	Yes
Did the case series have consecutive inclusion of participants?	Unclear	Unclear	Unclear
Did the case series have complete inclusion of participants?	Unclear	Unclear	Unclear
Was there clear reporting of the demographics of the participants in the study?	Yes	Yes	Yes
Was there clear reporting of clinical information of the participants?	Yes	Yes	Yes
Were the outcomes or follow up results of cases clearly reported?	Yes	Yes	Yes
Was there clear reporting of the presenting site(s)/clinic(s) demographic information?	Yes	Yes	Yes
Was statistical analysis appropriate?	Yes	Not applicable, descriptive analysis	Not applicable, descriptive analysis
Total score (out of 10 points) (Yes = 1, No = 0, Unclear = 0.5, N/A = 0)	9.0	8.0	8.0

*JBI Cohort Study Appraisal*	Mazzapicchi et al., 2022 (16)	Han et al., 2023 (26)	
Were the two groups similar and recruited from the same population?	Not applicable, single-cohort	Not applicable, single-cohort	
Were the exposures measured similarly to assign people to both exposed and unexposed groups?	Not applicable, single-cohort	Not applicable, single-cohort	
Was the exposure measured in a valid and reliable way?	Yes	Yes	
Were confounding factors identified?	Unclear	Unclear	
Were strategies to deal with confounding factors stated?	No	No	
Were the groups/participants free of the outcome at the start of the study (or at the moment of exposure)?	Yes	Yes	
Were the outcomes measured in a valid and reliable way?	Yes	Yes	
Was the follow-up time reported and sufficient to be long enough for outcomes to occur?	No	No	
Was follow-up complete, and if not, were the reasons to loss to follow-up described and explored?	Yes	Yes	
Were strategies to address incomplete follow-up utilized?	Not applicable, no incomplete follow-ups	Not applicable, no incomplete follow-ups	
Was appropriate statistical analysis used?	Yes	Yes	
Total score (out of 11 points) (Yes = 1, No = 0, Unclear = 0.5, N/A = 0)	5.0	5.0	

## 4 Discussion

### 4.1 Contrast-enhanced ultrasound overview

US imaging is an indispensable tool in spinal surgery, applied across a wide range of conditions, including degenerative diseases, trauma, deformities, and tumors (1, 2). Building on this foundation, CEUS has emerged as a novel modality that not only improves the visualization of vascular structures and tumor margins by leveraging the unique echogenic properties of microbubble contrast agents, but also utilizes the differential echogenicity between tumor tissue and normal spinal cord—particularly in cases where extradural and intradural tumors lack distinct margins—to confirm adequate decompression and guide complete resection (12, 13, 19, 20).

Crucially, CEUS addresses several key limitations of iMRI. While iMRI remains the gold standard, it provides only a static snapshot of the anatomy, which can be rendered inaccurate by cerebrospinal fluid loss and tissue shift (‘brain shift’) after dural opening (6–8). Furthermore, iMRI significantly prolongs operative time and requires expensive, specialized infrastructure (7). In contrast, CEUS offers a real-time, dynamic assessment of the resection bed that is unaffected by tissue shift, easily repeatable throughout the surgery, and cost-effective to implement in standard operating suites.

CEUS enables detailed visualization of tissue perfusion by using microbubble contrast agents that are less than 10 microns in diameter—small enough to navigate capillaries without extravasating into surrounding tissues (21). When the ultrasound probe emits a frequency that resonates with these microbubbles, they cyclically expand and contract, generating echoes that are captured by the probe’s piezoelectric crystals (22). This process produces a non-linear echo signal that is distinct from the surrounding tissue, thereby enhancing the signal-to-noise ratio and delineating perfused soft tissue structures with precision (23). Such enhanced imaging is potentially instrumental in confirming adequate decompression and ensuring complete tumor resection (24).

This capability, combined with the established use of conventional ultrasound in managing degenerative, traumatic, and deformity-related spinal conditions, underscores the growing application and clinical impact of CEUS in spinal oncology over the past five years (1, 2, 9, 16). Thus, this narrative review of available literature details the studies that have utilized CEUS to aid in spinal tumor dissection. Specially, we highlight the advantages CEUS brought to each case and summarize tumor characteristics observed on CEUS for the various pathologies.

### 4.2 Intradural intramedullary tumors

Vetrano et al. reported one of the first applications of CEUS in spinal tumor resection in a 14-year-old patient with suspected diffuse infiltrative intramedullary lesions (19). Preoperative MRI with gadolinium revealed three nodular lesions at C7, T1, and T4, all with cystic capsules and diffuse enhancement between them. The radiological findings initially suggested a tumor matching the characteristics of an astrocytoma. However, intraoperative CEUS revealed three cystic lesions without any enhancement in the intramedullary space between the lesions, differing from the preoperative MRI. CEUS also displayed a vascular peduncle located on the C7 lesion, protruding interiorly. These characteristics obtained by CEUS suggested a diagnosis of hemangioblastoma, which was later confirmed by pathology. Moreover, CEUS delineated the perfusion patterns of each lesion, allowing the surgical strategy to be modified for complete resection.

In a case series of 14 patients, Han et al. used CEUS to aid in the resection of 4 astrocytomas, 5 ependymomas, 1 anaplastic astrocytoma, and 4 cavernous hemangiomas (25). The authors stated that CEUS greatly improved visualization of tumor margins and perfusion patterns, which limited the extent of the initial durotomy. Such findings were especially true for the ependymomas and astrocytomas, which are generally poorly delineated. Of note, the authors found that there were varying degrees of enhancement of cavernous hemangiomas on CEUS; however, no further details of tumor characterization on CEUS were reported. Regardless, CEUS still allowed for the differentiation between cystic and solid tissues, displaying as hypoechoic and hyperechoic, respectively. After each resection, the authors performed CEUS imaging a second time to identify any residual tumors, confirming GTR along with favorable neurological outcomes at a mean follow-up of 15.9 months. These findings underscore CEUS’s additional value in detecting residual tumor. While long-term recurrence data in these specific studies is limited, the ability of CEUS to facilitate GTR, a well-established prognostic factor for progression-free survival, suggests a theoretical potential to reduce recurrence risk (1, 2, 4, 5).

Barkley et al. characterized several types of intramedullary tumors on CEUS in a seven patient case series consisting of three hemangioblastomas, two ependymomas, one subependymoma, and one pilocytic astrocytoma (9). Both hemangioblastomas and ependymomas displayed considerable contrast enhancement on CEUS, while there was a lack of contrast enhancement when imaging the pilocytic astrocytoma and subependymoma. However, the authors noted that even without contrast enhancement of the tumors, specifically within the pilocytic astrocytoma, the physiological uptake of contrast agent in the surrounding healthy tissue alone, which was starkly different from the tumor, sufficiently delineated the tumor margins. Additionally, in the hemangiomas, identifying the vascular supply with CEUS allowed for early coagulation and minimized intraoperative blood loss. CEUS also improved the delineation of the vascular pedicle of the ependymoma from the adjacent spinal cord tissue. Similar to Han et al., CEUS imaging was performed a second time, after each resection, indicating total resection was achieved (25). Overall, the authors demonstrated that intraoperative CEUS aided in resection of all of the reported tumors, especially the more vascularized tumor types, even without full contrast enhancement present.

In 2021, Vetrano and colleagues published an additional case series that further characterized intramedullary tumors on CEUS (3). Over a four-year period, the authors used CEUS to aid in the resection of two pilocytic astrocytomas, one anaplastic astrocytoma, one glioblastoma, four ependymomas, one subependymoma, two hemangioblastomas, and one neurocytoma. All tumors, except for the glioblastoma and anaplastic astrocytoma, displayed sharp margins on CEUS that delineated the tumor from surrounding spinal cord tissue. Additionally, the hemangioblastomas, glioblastoma, and anaplastic astrocytoma all rapidly enhanced upon contrast administration, whereas the pilocytic astrocytomas, subependymoma, and neurocytoma were slower to reach peak enhancement. The enhancement time of the four ependymomas varied from slow to rapid. Of note, a hypoechoic peritumor cyst in one of the ependymomas was found on CEUS, which contradicted the preoperative MRI findings and confirmed a syringomyelic dilation. In one pilocytic astrocytoma, CEUS findings suggested an expansion of the initial durotomy, which the authors noted was an influential step to achieve total resection. The authors were also able to capture tumor vascular patterns on CEUS that did not display any obvious patterns. This study suggested that the peak constant enhancement measured by CEUS may be related to the degree of vascularity within the tumor or arteriovenous shunts and venous engorgements that are uniquely influenced by mass effect and pathological angiogenesis. Furthermore, this study reinforces the concept that tumor delineation is inversely related to the invasive nature of the tumor type.

Mazzapicchi et al. used CEUS for resection of four intramedullary lesions as part of a comprehensive retrospective cohort on cranial and spinal resection imaging techniques for hemangioblastomas (16). All four hemangioblastomas were characterized as nodular and homogenously hyperechoic, with cystic components. Two of the tumors were noted to be deeply embedded. In such cases, CEUS helped with localization and surgeons were able to adapt the extent of the myelotomy in real-time. The CEUS complemented well with standard B-mode and Doppler imaging to provide dynamic guidance for resections. However, the authors note CEUS may be limited by a considerable learning curve for anatomical orientation, as well as the possibility of artifact creation by post-resection bleeding and/or hemostatic agents.

Lastly, an additional retrospective cohort study was performed by Han et al. that evaluated contrast perfusion patterns in 36 high grade gliomas: one pilocytic astrocytoma, seven low-grade gliomas, three anaplastic gliomas, seven glioblastomas, and 18 ependymomas (26). Overall, GTR was achieved with CEUS in a majority of cases, which was improved upon compared to previous resection reports at the author’s center (27). The visualization of tumor margins and cystic components was greatly enhanced by CEUS in the ependymoma and pilocytic astrocytoma cases. CEUS was also able to delineate low-grade gliomas, which generally have poor margins and enhancement on MRI. Additionally, while the parenchyma of the glioblastomas and anaplastic astrocytomas did not show obvious boundaries on CEUS, the high-grade gliomas displayed a higher degree of vascularization than surrounding tissues. High-grade gliomas also displayed a greater peak contrast intensity and were faster to reach peak intensity compared to low grade gliomas. While these findings were not statically significant, they agree with the diffusion patterns presented by Vetrano et al. and thus suggest a potential link between contrast perfusion patterns and tumor vascularity (3). CEUS was ultimately able to visualize the vascularization of the gliomas to guide resection and avoid unexpected blood loss or operative complications.

### 4.3 Intradural extramedullary tumors

Vetrano et al. demonstrated the advantages of CEUS compared to MRI and standard B-mode ultrasound imaging in the case of a 33-year-old female patient with dorsal Schwannoma (20). Although preoperative MRI suggested an intramedullary lesion, standard B-mode imaging showed a hyperechoic mass with poorly defined borders that did not clearly separate the tumor from the spinal cord. However, upon CEUS imaging, an extramedullary tumor was revealed, distinct from the surrounding perilesional cysts and the edematous spinal cord. Techniques were adjusted, and complete resection was confirmed by CEUS. The authors theorized that the combination of the perilesional cysts, considerable edema, and cord compression may be responsible for the intramedullary preoperative diagnosis. This case highlights the ability of CEUS to improve surgical planning and outcomes by characterizing spinal tumors to a high degree of specificity and by protecting against MRI misinterpretations.

Della Pepa et al. described the utility of using CEUS during resection of highly vascularized tumors. In one case specifically, a 61-year-old male with recurrent dorsal hemangiopericytoma, confirmed as an intradural extramedullary lesion, underwent a T12 laminectomy for resection (13). Here, real-time CEUS was pivotal in revealing the vasculature architecture of the tumor before durotomy. With visualization of the tumor’s feeder arteries and venous drainage, the authors reported a reduction blind surgical maneuvering, avoided damage to the displaced nerves, and achieved GTR. Furthermore, additional CEUS imaging following the initial dissection identified a residual tumor, which was removed and later confirmed to be pathologic. This case demonstrates how the visualization of the vasculature by CEUS pre- and post-operatively can be a useful tool to ensure safe total resections in vascularized intradural extramedullary tumors.

### 4.4 Extradural extramedullary tumors

Extradural extramedullary tumors are outside of the dural layer, and to our knowledge, no published reports have described the use of CEUS for resection of extradural extramedullary tumors. However, based on insights gained from these intradural cases, we speculate that CEUS could be a valuable adjunct for visualizing vascular architecture and delineating tumor margins in extradural lesions, potentially aiding in achieving complete resection. For extradural masses, such as meningiomas, schwannomas, and chordomas, while often not involving the spinal cord, they often lead to nerve root compression. Like intradural masses, CEUS could allow valuable imaging that can help with safe resection without injuring adjacent nerve roots. Moreover, assessment of perfusion parameters acquired from CEUS can provide valuable insight into characteristics of the tumor and assist in diagnosis and prognosis. Much like its use in hepatology, where CEUS has been shown to have high specificity for diagnosing liver nodules as hepatocellular carcinoma, CEUS has the potential to provide diagnostic value to undiagnosed extradural masses such as metastatic processes and primary cancers of the spine. As we continue to utilize both ultrasound and CEUS for the spine and spinal cord, its application to extradural masses will surely be realized.

### 4.5 CEUS limitations

While such studies highlight the potential of CEUS within spine tumor resections, there are still limitations of the technique. First, despite the high resolution provided by modern high-frequency transducers, the physics of ultrasound waves results in limited penetration depth. This can impede the visualization of deeply seated ventral tumors or lesions obscured by complex bony anatomy, necessitating careful preoperative planning regarding the surgical window. The imaging scope is further restricted by the initial approach; the spinal cord must be exposed from the overlying laminae to provide access for the ultrasound probe. Therefore, an insufficient laminectomy may prevent full tumor visualization by CEUS.

Secondly, the application of intraoperative transducers entails technical challenges and potential risks related to their introduction into the surgical cavity. The physical footprint of the probe requires a sufficiently large laminectomy for adequate contact (3, 20), and the manipulation of the device in close proximity to the exposed spinal cord carries a risk of mechanical injury, requiring a steady hand and significant operator experience.

A third significant drawback is the occurrence of acoustic artifacts. Acoustic enhancement artifacts, often caused by the difference in signal attenuation between the physiological saline in the resection cavity and the surrounding spinal tissue, can create hyperechoic noise. As Mazzapicchi et al. noted, these artifacts can be observed from post-resection bleeding or with the presence of hemostatic agents (16). These artifacts may obscure residual neoplastic fragments, particularly along the resection cavity walls, thereby complicating the assessment of GTR.

Furthermore, standard CEUS relies on the interpretation of 2D cross-sectional images, which can be difficult to mentally reconstruct into a 3D volume, especially within a confined surgical approach. Unlike iMRI, there is currently a lack of commercially available 3D neuronavigation systems fully integrated with CEUS. This absence diminishes the ability to precisely map the volumetric extent of diffuse or infiltrative tumors in stereotactic space.

Moreover, CEUS is highly operator-dependent. The acquisition of high-quality images and the real-time interpretation of perfusion patterns require a specialist proficient in ultrasound diagnostics. This learning curve regarding anatomical orientation and image interpretation may hinder the widespread adoption of the technique in centers without dedicated training or access to an experienced sonographer within the surgical team (3, 16, 26).

Finally, the benefits of CEUS for tumor margin visualization may be limited by tumor pathology. As previously mentioned, margins of high-grade glioblastomas and anaplastic astrocytomas were reported to be poorly visualized in some studies (3, 25, 26). However, recent publications assessing the efficacy of transcutaneous CEUS to evaluate the spinal cord have shown promise, suggesting a future strategy that could allow for serial assessment of tumor recurrence without undergoing expensive MRI assessment (28, 29).

### 4.6 Study limitations

To our knowledge, this is the first review to examine the use of CEUS in spinal tumor resections. However, several study limitations should be acknowledged. The review included a majority of case reports and case series, which limited the population size and increases risk of selection bias. The non-random nature of case reports and series also limits this study’s ability to form comparative conclusions. Furthermore, the included studies were limited in data on patient outcomes. Most follow-up lengths were either not reported or less than 3 months, arguably an inadequate amount of time to observe long-term adverse outcomes. There were no objective measures, such as tumor reoccurrence or development of neurological injury, provided to quantify the success of tumor resection. Without comprehensive individual patient data, this study was limited to a narrative review.

## 5 Conclusion

CEUS is a dynamic intraoperative imaging tool that has been used to assist spinal tumor resections in tens of patients over the past decade. In such cases, CEUS was able to improve the visualization of tumor margins and characterize perfusion patterns to guide spinal tumor resection. These findings can be used to guide tumor characterization in future CEUS-assisted resections. However, the current body of literature surrounding CEUS in spinal tumor resection mainly contains case reports and case series, most of which were tumor located intradural intramedullary. Therefore, there is a need for further research into its applications. Additionally, improvement in ultrasound technology to better suit the unique challenges of spine surgeons promises to further establish its value in spinal oncology.
